# Editorial: The Role of Modern Neuro-oncology in the Treatment of Primary CNS Tumors, and Brain and Spinal Metastases

**DOI:** 10.3389/fonc.2020.00759

**Published:** 2020-06-03

**Authors:** Marcos Maldaun, Sujit Prabhu, Claudio Tatsui, Luis Souhami

**Affiliations:** ^1^IEP Hospital Sírio-Libanês, São Paulo, Brazil; ^2^University of Texas MD Anderson Cancer Center, Houston, TX, United States; ^3^McGill University Health Centre, Montreal, QC, Canada

**Keywords:** neuro-oncology, CNS tumors, brain metastases, spinal metsatases, gliomas, meningeomas

This special multi-disciplinary article collection presents an exciting summary of what is new, promising, and controversial in the neuro-oncology field. Despite major advances in brain and spine tumor research as well as important developments and progress made in treatment delivery, unlike some other malignancies, we are unfortunately still far away from achieving a cure or even significantly improving survival rates in most pathologies in the neuro-oncology domain. On the other hand, many new developments in several areas in neuro-oncology have lately been reported, including new imaging technology, a better molecular and genetic pathology understanding, newer and safer surgical techniques, and promising target-oriented immunotherapy and chemotherapy strategies. In this special edition of Frontiers Neuro-Oncology, several of these Research Topics are presented, bringing new hope for improving outcomes in many areas.

Over the last 20 years, advances in surgery, radiation oncology, and systemic therapy associated with a better understanding of molecular and genetics profile in glioblastoma has lead to an improvement in median survival from 9 to 24 months. Although this is a significant change, it is clear that further improvement is necessary. Chaddad et al., in a comprehensive review, discuss how radiomics may aid in building predictive models for diagnosis, prognosis, and therapeutic response. Daniel et al. also review potential reasons for treatment resistance post-temozolomide treatment and describe the *hypermutant* genomic outcome (temozolomide-induced) and its possible impact on disease recurrence and newer therapeutic approaches. Del Bene et al. describe how useful advanced intra-operative ultrasound can be by guiding the surgeon to achieve a maximal safe resection in real time. On the same line, Chowdhury et al. report on a systematic review of the role of intra-operative MRI in awake neurosurgical procedures, pointing out that it is an imaging technique feasible and safe to perform.

In the pituitary field, Waddle et al., using validated instruments, for the first time characterized, in a prospective fashion, usual symptoms, complications, and quality of life before and after the sub-acute period of post-surgical intervention. A disturbed sleep pattern was identified as the culprit for worsened quality of life in such patients. Of interest, Guo et al. describe novel mutant genes in a patient with pituitary carcinoma. The authors were able to identify an uncommon P53 mutation in addition to novel mutations in ATRX and PTEN genes. This discovery can lead to important therapeutic changes by targeting very specific mutations. It may also help our understanding of the innate biology of these tumors.

Health-related quality of life (HRQOL) metrics are unequivocally a very important aspect of treatment outcomes for all neuro-oncological disorders and a growing concern among investigators. There is now an increasing interest toward incorporating measurements of quality of life as endpoints in most studies. Two reports in this collection deal with this aspect of patients' management. Gabel et al. report the first use of PROMIS and NEURO-QOL, two validated questionnaires, to assess functional domain that could alter HRQOL in adult patients with low- and high-grade gliomas. The authors show that low-grade gliomas patients experience more pain intensity and greater distress compared to high-grade patients, while the latter do develop more significant neuro cognitive dysfunction. They also demonstrate that these two HRQOL tools are valuable questionnaires. From another front, Cacho-Díaz et al. from Mexico used a Mexican-Spanish version of the QLQ-BN20 instrument in patients with primary or metastatic brain tumors and showed its validity and reliability. Of importance is that the authors studied a bivariate association of HRQOL and overall survival, and they report that several domains affecting HRQOL were associated with a poorer overall survival. These findings challenge us to utilize patient reported outcomes metrics as measures to facilitate specific interventions.

Lastly, Suki et al., from the M.D. Anderson Hospital, describe a structured database from their institution that provides extremely useful guidelines for other investigators who are initiating such process in their centers. This large repository of clinical information has allowed them to obtain significant resources over time leading to hundreds of publications including landmark papers and practical and helpful information for the scientific community all over the world. Big data may further elucidate unknown mechanisms of chemo-resistance, for example, and will hopefully help in developing opportunities for novel treatments.

We hope the papers reported in this special collection (Ballester et al.;Cacho-Díaz et al.; Chaddad et al.; Chowdhury et al.; Daniel et al.; Del Bene et al.; de Oca Delgado et al.; Gabel et al.; Guo et al.; Ian Robins et al.; Suki et al.; Waddle et al.; Wang et al.) will aid investigators in their daily battle to improve outcomes in patients harboring nervous system tumors. We all appreciate how heterogeneous disease of the nervous system can be, and we agree that individualizing treatment based on clinical and pathological parameters remains a valid strategy in many situations. The modern neuro-oncology must integrate findings from the bench, including development of new systemic agents, to newer technological surgical and radiotherapy advances. We firmly believe this collection describes novel treatment paradigms and highlights the need for a multi-disciplinary neuro-oncological approach that emphasizes good perspectives for better and solid results in the management of nervous system tumors.

## Author Contributions

All authors listed have made a substantial, direct and intellectual contribution to the work, and approved it for publication.

## Conflict of Interest

The authors declare that the research was conducted in the absence of any commercial or financial relationships that could be construed as a potential conflict of interest.

